# Recent Advances in Electrocatalytic Treatment and Valorization of Pulping and Papermaking Wastewater

**DOI:** 10.3390/molecules31101604

**Published:** 2026-05-11

**Authors:** Yuchen Bai, Shuangshuang Liu, Xiangchi Liu, Xuebing Zhao

**Affiliations:** 1 Beijing Key Laboratory of Flavor Chemistry, Beijing Technology and Business University, Beijing 100048, China; 2 School of Food and Health, Beijing Technology and Business University, Beijing 100048, China; 3 Key Laboratory of Industrial Biocatalysis, Ministry of Education, Tsinghua University, Beijing 100084, China; 4 Institute of Applied Chemistry, Department of Chemical Engineering, Tsinghua University, Beijing 100084, China

**Keywords:** pulping and paper-making wastewater, pollutant composition, electrochemical, resource utilization

## Abstract

The pulping and paper-making (P&P) industry is one of the world’s largest manufacturing sectors, yet it is plagued by high water/energy consumption and massive discharge of highly polluted wastewater. The effluents from pulping, bleaching and papermaking processes are characterized by high chemical oxygen demand (COD), intense color, toxic adsorbable organohalides (AOX) and abundant refractory lignin, which pose significant threats to aquatic ecology and human health. Although conventional physical, chemical and biological treatments have been widely applied, they are constrained by insufficient degradation efficiency toward recalcitrant organics, high cost and potential secondary pollution. In recent years, electrocatalytic technologies including electrocatalytic oxidation, electroreduction and their integrated processes, have demonstrated superior efficacy in specific scenarios of P&P wastewater treatment, such as lignin degradation, toxic side-streams treatment, pretreatment for enhancing biodegradability, and polishing steps in integrated treatment systems, which are not universally applicable solutions for P&P wastewater remediation. Meanwhile, biomass fuel cells typified by direct biomass fuel cells (DBFC) and microbial fuel cells (MFC) provide promising pathways for synchronous pollutant removal, energy production and resource recovery. Representative studies have reported COD removal efficiencies of 60–100% for electrochemical and advanced oxidation processes, while integrated electro-Fenton–biological treatment increased the BOD/COD ratio from 0.34 to 0.52 and achieved an overall COD removal of 94%. It should be noted that these advanced electrochemical technologies are still confronted with challenges in industrial scale-up, high energy and electrode material costs, and stable continuous operation. This review systematically elaborates on the physicochemical properties, generation mechanisms and environmental impacts of P&P wastewater, comprehensively summarizes the mainstream treatment technologies including physicochemical, biological, electrochemical and integrated processes, and analyzes their reaction mechanisms, efficiencies and applicable conditions. Particular emphasis is placed on electrocatalytic treatment and bio-electrochemical valorization strategies. This review is anticipated to provide a valuable reference for the efficient and targeted treatment as well as sustainable utilization of P&P wastewater, thereby supporting the green and low-carbon development of the P&P industry.

## 1. Introduction

**Figure 1:**
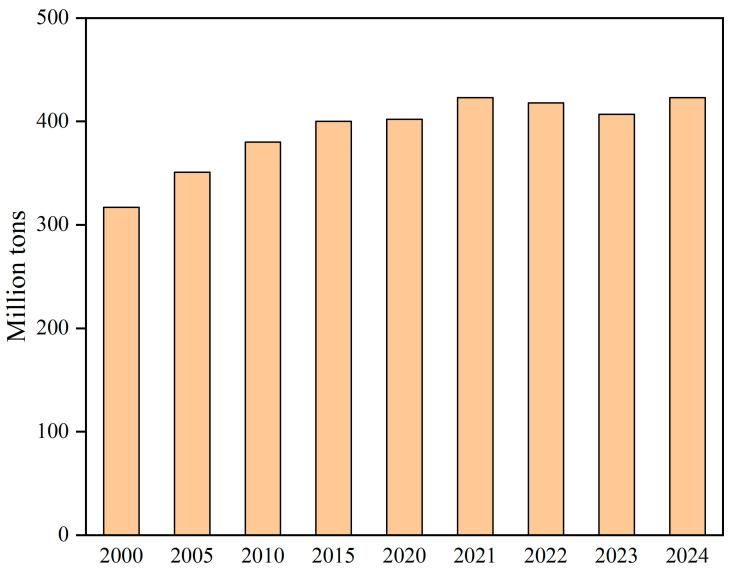
Global paper and paperboard annual production in the years 2000 to 2024, according to FAOSTAT [2].

The pulping and paper-making (P&P) industry has become one of the largest industries in the world due to the huge demand for paper and its related products. As shown in Figure 1, global paper and paperboard production remained at a high level during 2000–2024 and reached 423 million tons in 2024, according to FAOSTAT [1,2]. Meanwhile, the P&P industry is characterized by high water and energy consumption, as well as the generation of massive volumes of highly polluted wastewater. In the traditional P&P process, the manufacture of 1 ton of paper may emit approximately 950 kg of CO_2_. At the industry level, the P&P sector has been reported to account for about 5.7% of global final industrial energy consumption and about 9% of manufacturing-related greenhouse gas emissions. The P&P industry is ranked as the world’s third largest consumer of freshwater [3]. The primary sources of wastewater include the cooking, bleaching, and washing stages, generating streams such as black liquor (BL), mid-stage water, and white water [4]. The water footprint of a P&P plant is substantial, typically ranging from 5 to 100 m^3^ per ton of paper produced, depending on factors such as the raw material, paper grade, and water recycling rate. Given the environmental pressures associated with P&P production processes, the integration of production and environmental protection has become a core focus in the industry. According to statistics from the Food and Agriculture Organization (FAO), the annual wastewater discharge from China’s paper industry alone reaches as high as 4 billion cubic meters [5]. In the United States, it is considered the third-largest polluter, while an estimated 50% of all waste discharged into Canadian waters is attributed to this industry [6]. In developing countries, challenges such as low water reuse rates and inadequate wastewater management lead to the generation of massive wastewater volumes. This effluent often constitutes nearly 10% of national industrial discharge and is characterized by extremely high concentrations of chemical oxygen demand (COD) and total suspended solids (TSS), causing severe damage to aquatic ecosystems. BL contains high concentrations of lignin, hemicellulose, and alkali metal salts [7]. Its direct discharge triggers water eutrophication, toxic substance accumulation, and even the formation of carcinogens like dioxins, leading to the death of aquatic organisms and disrupting terrestrial ecosystems [8,9]. Bleaching wastewater is characterized by the presence of adsorbable organic halides (AOX), many of which are highly mutagenic and carcinogenic. Washing wastewater is typically rich in suspended solids as well as high concentrations of dissolved and colloidal substances (DCS) [10]. More than 250 chemicals have been identified in effluents produced at different stages of papermaking [11]. Kumar and Verma conducted a comprehensive evaluation of hazardous emissions originating from this industry, with particular emphasis on their potential adverse impacts on human health [12]. Collectively, these issues place immense environmental pressure on the global P&P industry, highlighting an urgent need for advanced pollution control strategies to safeguard ecological safety.

Nowadays, the P&P industry faces challenges in complying with stringent environmental regulations. In recent years (2020–2026), global regulations on pulp and paper wastewater have become increasingly stringent. Discharge limits for COD, AOX, TSS, total nitrogen, and total phosphorus have been lowered, while emerging contaminants—such as dioxins, aniline, and estrogenic compounds—are now subject to toxicity-based criteria. In China, the updated GB 3544 standard and the “14th Five-Year Plan” policies enforce stricter concentration and total emission limits, mandate water reuse, and promote cleaner production. In the EU and North America, revised BAT documents and effluent regulations emphasize ECF/TCF bleaching, source reduction, and circular water management. These regulatory updates have rendered many previous treatment evaluations obsolete, underscoring the urgent need for advanced, regulation-compliant treatment technologies, which constitute the central focus of this review.

Significant research efforts and policy initiatives, such as those supported by the European Commission, have been undertaken to minimize the generation of P&P wastewater, encouraging the adoption of cleaner production technologies aimed at reducing pollutant loads at the source. Despite extensive research into P&P wastewater treatment strategies, a universally accepted best practice has yet to be established. Conventional treatment methods, such as landfill and incineration, can lead to potential secondary environmental impacts, including leachate formation and the emission of harmful gases, which may further exacerbate the environmental burden [13]. Currently, industrial wastewater management in the P&P industry relies primarily on a combination of physical, chemical, biological, and hybrid treatment processes. Physical techniques used as pretreatment, such as sedimentation and filtration, are effective at removing suspended solids and colloidal substances. The main treatment stage aims at degrading high-concentration organic pollutants (COD up to 3000–18,000 mg/L). Conventional technologies include biological treatment (e.g., activated sludge process, multi-stage AO process) and physical-chemical treatment; for high-concentration waste liquor (e.g., BL), anaerobic reactors (e.g., IC reactor) are often used to degrade organic matter and produce biogas for energy recovery. The advanced treatment stage is designed to remove refractory pollutants and meet strict discharge limits, with common technologies including advanced oxidation processes (AOPs, e.g., Fenton fluidized bed), membrane separation (e.g., ultrafiltration, reverse osmosis), and DTRO membrane concentration technology. However, significant challenges still persist in the current treatment of P&P wastewater, such as excessive solvent consumption, high operating costs, the risk of secondary pollution, and sensitivity to inhibitory compounds, which are commonly found in P&P wastewater.

Recently, electrochemical methods have emerged as a promising alternative for P&P wastewater treatment, rather than a universally applicable solution. This technology encompasses direct and indirect oxidation, reduction, and electro-Fenton processes [14]. It exhibits multiple advantages, including high removal efficiency for refractory pollutants such as lignin and phenol compounds, as well as strong potential to complement traditional biological methods in treating recalcitrant organic matter. Notably, these electrochemical technologies are most suitable for specific application scenarios, including the treatment of refractory or toxic side streams, pretreatment to enhance biodegradability, and polishing steps in integrated treatment systems. Moreover, electrochemical methods are regulated by parameters such as current, voltage, electrode material, and reaction time, demonstrating high process flexibility and controllability. Combinations of electrochemical and biological methods can even reduce energy consumption and enable resource recovery [15]. However, these advanced electrochemical technologies still face considerable challenges in industrial application, including difficulties in scale-up from laboratory to industrial scale, high energy and electrode material costs, and limitations in stable continuous operation. Additionally, in chloride-containing P&P wastewater systems, electrochemical processes may induce the formation of hazardous by-products (e.g., chlorinated compounds and elevated AOX levels), which pose additional risks to environmental safety and downstream treatment processes.

Considering the above points, this review aims to clarify the characteristics and key pollutants of wastewater generated by the P&P process, with particular attention to recalcitrant components such as lignin and AOX, as well as the pollution challenges associated with different wastewater streams. It further provides a systematic overview of the principles, treatment performance, advantages, and limitations of conventional and emerging P&P wastewater treatment technologies, with particular emphasis on electrocatalytic processes and their integrated applications for high-strength and refractory effluents, while explicitly addressing their specific applicability, industrial maturity, and potential by-product risks. In addition, this review discusses the potential of biomass fuel cell technologies for the treatment and valorization of P&P waste streams, with the aim of clarifying both the opportunities and the practical constraints associated with environmentally sound treatment and resource recovery in the P&P industry.

## 2. Pulping and Paper-Making Processes and Wastewater Characteristics

**Figure 2:**
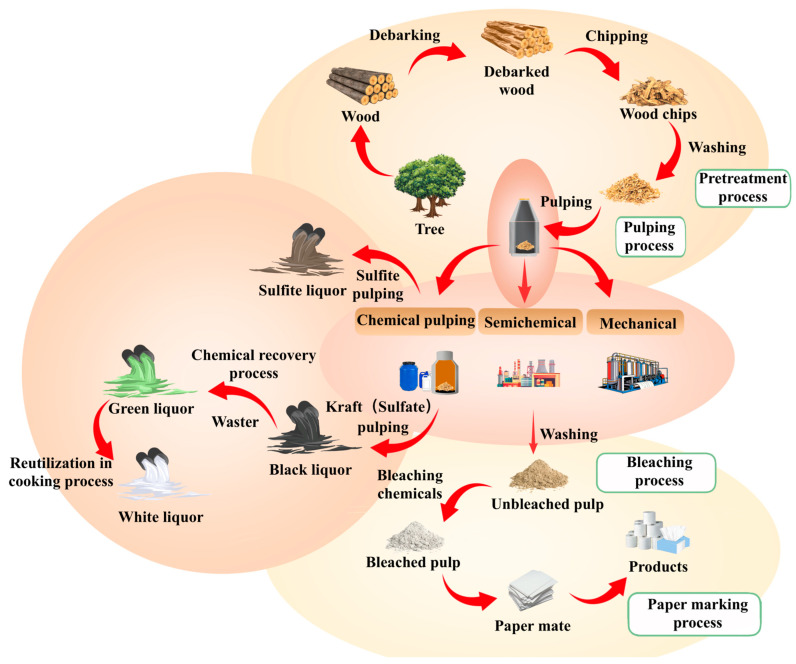
Pulping and paper-making process flow.

The pulping and paper-making process is a complex multi-stage system that converts lignocellulosic raw materials into value-added products [16]. It typically consists of four stages: raw material preparation, pulping, pulp treatment (including washing, screening, and bleaching), and papermaking [17]. In the first step, the raw materials undergo preparation, debarking, and chipping before entering the pulping process. Different pulping methods are then applied. Bleaching is subsequently carried out to improve the quality and brightness of the brown pulp. After that, the treated pulp is dewatered and dried to obtain the final product. A more detailed schematic of production stages in the P&P industry is shown in Figure 2. Each stage involves specific chemical and physical operations, which directly determine the composition, concentration, and toxicity of the generated wastewater. The systematic classification of pulping processes and the comparative analysis of their wastewater characteristics are essential for optimizing pollution control strategies and promoting clean production in the industry [18].

### 2.1. Pulping Processes and Wastewater Characteristics

Pulping represents the fundamental process for liberating cellulose fibers from lignocellulosic feedstocks and can be broadly categorized into three principal types according to the underlying fiber separation mechanisms: chemical pulping, mechanical pulping, and semi-chemical pulping. The choice of pulping technology is largely governed by the nature of the raw materials, the targeted product performance, and the balance between economic feasibility and environmental considerations, resulting in substantial variations in operational conditions, fiber yield, and the quantity and characteristics of generated pollutants. It is the largest source of pollution in the whole process of papermaking. Large amounts of wastewater are generated at different stages.

#### 2.1.1. Chemical Pulping

Chemical pulping involves the selective solubilization of lignin that binds cellulose fibers within the wood matrix, thereby enabling the effective separation of individual fibers. Lignin depolymerization and dissolution are achieved through the combined effects of elevated temperature and pressure in the presence of chemical reagents. Among chemical pulping technologies, the kraft (or soda) process and the sulfite process are recognized as the two predominant industrial methods.

The kraft process, first patented by Carl Ferdinand Dahl in 1884, marked a major advancement in pulp manufacturing through the implementation of sulfate-based cooking chemistry [19]. In this process, wood chips are subjected to digestion at elevated temperatures (150–170 °C) and pressures (6–7 atm) in a cooking solution commonly referred to as white liquor, which mainly consists of sodium hydroxide (NaOH) and sodium sulfide (Na_2_S). Under these strongly alkaline conditions, ether linkages within the lignin macromolecular structure are cleaved, promoting lignin depolymerization and subsequent dissolution. Approximately 55% of the wood mass, including lignin, polysaccharides, and extractives, is dissolved into the pulping liquor, resulting in two primary products: cellulose pulp (yield: 45–50%) and the spent cooking liquor, known as BL. BL is a complex, viscous aqueous solution containing alkali lignin, lignin fragments, carboxylic acids, resins, and residual inorganic chemicals.

A distinctive advantage of the kraft process is its closed-loop chemical recovery system, wherein inorganic compounds are regenerated from the BL in a recovery boiler, while organic residues are incinerated for energy [19]. This system significantly reduces chemical consumption and pollutant emissions. It has superseded the conventional soda pulping method, which relies solely on NaOH, due to several distinct advantages: its applicability to all wood species, a robust chemical and energy recovery system, and the production of pulp with superior strength. However, kraft pulping does not completely remove lignin, necessitating a subsequent bleaching stage using oxidizing agents to degrade residual lignin and achieve high pulp brightness [20]. The evaporation of BL also generates a condensate rich in methanol and reduced sulfur compounds, which requires targeted treatment [21].

BL is the dominant wastewater stream from kraft pulping, accounting for over 90% of the total organic pollutant load of the kraft pulping process. It is a complex, viscous alkaline solution with a pH value of 9–12, mainly containing alkali lignin, lignin degradation fragments, hemicellulose degradation products (e.g., formic acid, acetic acid), wood extractives (resins, fatty acids), and residual inorganic chemicals (NaOH, Na_2_S) [22]. The key pollutant indicators of BL are extremely high: the COD concentration reaches tens of thousands of mg/L, and the biodegradability is poor (biochemical oxygen demand (BOD)/COD ratio: 0.15–0.3) due to the high content of recalcitrant lignin derivatives, which are difficult to be degraded by microorganisms [23].

In addition to BL, the evaporation condensate of BL is another critical wastewater stream in the kraft pulping process. The condensate stream is characterized by elevated concentrations of volatile organic compounds (VOCs), including methanol, ethanol, furfural, and terpenes, together with total reduced sulfur (TRS) species such as hydrogen sulfide (H_2_S) and methyl mercaptan (CH_3_SH) [24]. Although this stream represents only approximately 5% of the total wastewater volume generated in pulp mills, it can account for 5–20% of the overall biochemical oxygen demand (BOD) load, thereby contributing disproportionately to odor emissions and posing considerable risks related to biological toxicity [25]. In addition, the bleaching stage of kraft pulp production—commonly employing multi-stage elemental chlorine-free (ECF) sequences such as O–D–E–D (oxygen delignification–chlorine dioxide bleaching–alkaline extraction–chlorine dioxide bleaching)—produces bleaching effluents enriched with adsorbable organic halogens (AOX), residual oxidizing agents, and lignin-derived oxidation products. These constituents are typically characterized by high toxicity and strong resistance to biological degradation [10].

The conceptual basis for producing delignified pulp from wood through sulfite pulping was first established by Benjamin C. Tilghman, who patented the process [26]. In sulfite pulping, lignin is solubilized under acidic conditions (typically pH 1–2) through the action of active sulfite species, including calcium, magnesium, ammonium, or sodium sulfites, resulting in the formation of water-soluble lignosulfonic acids [27]. In comparison with the kraft process, sulfite pulping is generally conducted under relatively milder temperature and pressure conditions and yields pulp with higher brightness but reduced mechanical strength. The pulp yield typically falls within the range of 50–60%, slightly exceeding that of kraft pulping, although the exact yield is strongly influenced by the nature of the lignocellulosic feedstock and the applied cooking parameters [28].

The spent liquor generated from sulfite pulping, commonly referred to as spent sulfite liquor (SSL), exhibits markedly different composition and characteristics compared to BL. SSL is a complex acidic wastewater, containing carbohydrates that predominantly exist as monosaccharides under strong acidic conditions, whereas in bisulfite wastewater, oligosaccharides and polysaccharides are more prevalent. Sulfur occurs in multiple forms, including free sulfur dioxide (SO_2_), loosely bound SO_2_ in the form of hydroxysulfonic acids, and sulfonated species such as lignosulfonates [29]. Degradation products derived from lignin and hemicellulose account for a substantial fraction (25.9%) of the dissolved solids [30]. Moreover, SSL contains high concentrations of inhibitory compounds, estimated at 50.6%, encompassing dissolved solids, residual sulfites, wood extractives, organic acids, phenolic compounds, furan derivatives (e.g., furfural and 5-hydroxymethylfurfural), and lignin sulfonates [27].

#### 2.1.2. Mechanical Pulping

Mechanical pulping separates wood fibers through physical pressure, typically by pressing debarked logs against rotating millstones. This action abrades the wood, thereby liberating fibers that are subsequently suspended in water. This process achieves an ultra-high yield (85–95%), as most of the wood components (lignin, hemicellulose, and cellulose) are retained in the pulp. Due to the high residual lignin content, mechanical pulp has lower strength and poorer dimensional stability compared to chemical pulp, limiting its application to low-strength paper products (e.g., newsprint, tissue paper). To compensate for strength deficiencies, mechanical pulp is often blended with chemical pulp. The primary advantage of mechanical pulping is its low chemical consumption and reduced pollutant emissions, but the high lignin content requires bleaching (using hydrogen peroxide or sodium dithionite) to avoid fiber damage, which still generates a certain amount of bleaching wastewater [31].

The pollutant load of mechanical pulping wastewater is significantly lower than that of chemical pulping wastewater, and its pollution sources are mainly concentrated in the wood washing and pulp washing stages. The main pollutants include suspended solids (fibers, fiber fines), dissolved organic matter (lignin fragments, wood extractives), and small amounts of resin acids, fatty acids, and residual bleaching agents (e.g., peroxides). The COD concentration of mechanical pulping wastewater is typically 1000–3000 mg/L, and the BOD/COD ratio is 0.3–0.4, which is significantly higher than that of chemical pulping wastewater, indicating better biodegradability [32]. The bleaching effluents of mechanical pulp have low AOX concentrations but contain residual peroxides and lignin oxidation products. Overall, the pollutant emission intensity of mechanical pulping is only 1/5–1/3 of that of chemical pulping, making it a relatively environmentally friendly pulping technology [33].

#### 2.1.3. Semi-Chemical Pulping

Semi-chemical pulping is a hybrid approach, which employs milder chemical treatment (e.g., lower temperatures, more dilute cooking liquors, or shorter cooking times) followed by mechanical refining to separate the fibers. A common digestion step involves heating wood chips in a solution of sodium sulfite (Na_2_SO_3_) and sodium carbonate (Na_2_CO_3_) to partially remove or soften lignin, followed by mechanical refining to separate fibers. The yield of semi-chemical pulping ranges from 55% to 90%, depending on the specific method employed [34]. 

Common semi-chemical pulping processes include the two-stage vapor process. The resulting pulp has higher strength than mechanical pulp and higher yield than chemical pulp, making it suitable for medium-strength paper products (e.g., corrugated board, packaging paper and tissue paper). However, the high residual lignin content (15–25%) in semi-chemical pulp increases the difficulty of subsequent bleaching and contributes to the complexity of wastewater composition [35].

The wastewater characteristics of semi-chemical pulping are intermediate between chemical pulping and mechanical pulping. The primary wastewater streams include spent cooking liquor, pulp washing wastewater, and bleaching effluents. The spent cooking liquor contains partially dissolved lignin, hemicellulose degradation products, and residual chemicals (sodium sulfite, sodium carbonate), with a COD concentration of 5000–15,000 mg/L [36]. The washing wastewater has high suspended solids content (fibers, uncooked wood chips) and DCS. Due to the high residual lignin content in semi-chemical pulp, the bleaching effluents contain residual lignin and small amounts of AOX. The total pollutant load of semi-chemical pulping is 1.2–1.8 times that of mechanical pulping, but 40–60% lower than that of chemical pulping [37,38].

To reflect the differences in wastewater characteristics among different pulping processes, the key indicators of wastewater from chemical pulping (kraft, sulfite), mechanical pulping, and semi-chemical pulping are summarized in Table 1.

### 2.2. Bleaching Processes and Wastewater Characteristics

Bleaching is a crucial post-pulping treatment to improve pulp brightness and optical properties. It is achieved by cleaving conjugate double bonds in lignin chromophores through oxidation or reduction, thereby removing residual lignin. The selection of bleaching processes and agents is jointly determined by pulp type (residual lignin content), final product quality requirements, and environmental regulations, which directly govern the composition, toxicity, and treatability of the resulting bleaching wastewater [32].

Bleaching processes are categorized into three types based on the nature of bleaching agents: chlorine-containing bleaching (CCF), ECF bleaching, and totally chlorine-free (TCF) bleaching. Traditional chlorine-containing bleaching uses molecular chlorine (Cl_2_) as the core agent, featuring high efficiency and low cost but generating substantial toxic organochlorine compounds (e.g., dioxins, chlorinated phenols), which pose severe ecological risks and have been gradually phased out in industrial applications [8].

ECF bleaching, the current mainstream technology for chemical pulp, replaces elemental chlorine with chlorine dioxide (ClO_2_), supplemented by oxygen (O_2_) and hydrogen peroxide (H_2_O_2_). This process reduces AOX emissions by 80–90% compared to chlorine-containing bleaching while ensuring pulp strength and brightness, making it the preferred choice for large-scale pulp production. TCF bleaching completely abandons chlorine-based agents, relying on oxygen, hydrogen peroxide, ozone (O_3_), and peracetic acid. It achieves zero AOX emissions but requires higher agent dosage and stricter process control, resulting in relatively high operational costs [39].

Bleaching sequences are tailored to pulp types. For chemical pulp with low residual lignin (5–10%), multi-stage sequences such as O-D-E-D (oxygen delignification-chlorine dioxide bleaching-alkaline extraction-chlorine dioxide bleaching) are adopted to achieve high brightness [40]. For mechanical and semi-chemical pulps with high residual lignin (15–35%), gentle single or dual-stage sequences (e.g., P, P-Z) using hydrogen peroxide or sodium dithionite are employed to avoid fiber damage and maintain pulp strength [41].

Bleaching wastewater is an important component of P&P wastewater, with a complex composition and strong toxicity. Its characteristics are mainly determined by the bleaching process type, pulp type, and bleaching agent dosage, showing significant differences among different processes. CCF wastewater is strongly acidic (pH 2–4), with high AOX concentration (500–2000 mg/L). It contains a variety of toxic organochlorine compounds, such as dioxins, furans, and chlorinated phenols, which have strong bioaccumulation and carcinogenicity. The COD concentration of chlorine-containing bleaching wastewater is 3000–8000 mg/L, and the BOD/COD ratio is only 0.1–0.2, indicating poor biodegradability [41]. These pollutants pose serious risks to aquatic ecosystems and human health. The pH of ECF bleaching wastewater is weakly acidic to neutral (pH 5–7), and the AOX concentration is reduced to 50–300 mg/L, which is 80–90% lower than that of chlorine-containing bleaching wastewater. The main pollutants include lignin oxidation products (quinones, carboxylic acids), residual ClO_2_, and chlorides. The COD concentration is 1500–4000 mg/L, and the BOD/COD ratio is 0.2–0.3, with slightly improved biodegradability compared to chlorine-containing bleaching wastewater [3,42]. TCF bleaching wastewater is alkaline (pH 8–10) and contains almost no AOX. The main pollutants are lignin oxidation fragments, residual H_2_O_2_/O_3_, and organic acids (formic acid, acetic acid). The COD concentration is 1000–3000 mg/L, and the BOD/COD ratio is 0.2–0.3, showing good biodegradability [43]. However, due to the high dosage of bleaching agents and strict process control requirements, the treatment cost of TCF bleaching wastewater is relatively high. In addition, bleaching wastewater has the characteristics of high chroma (1000–5000 times), which is mainly derived from the unsaturated conjugated structures in lignin degradation products. The chroma of bleaching wastewater is difficult to remove by traditional biological treatment processes, and advanced treatment technologies such as advanced oxidation and adsorption are usually required for deep decolorization [44].

### 2.3. Papermaking Process and Wastewater Characteristics

**Figure 3:**
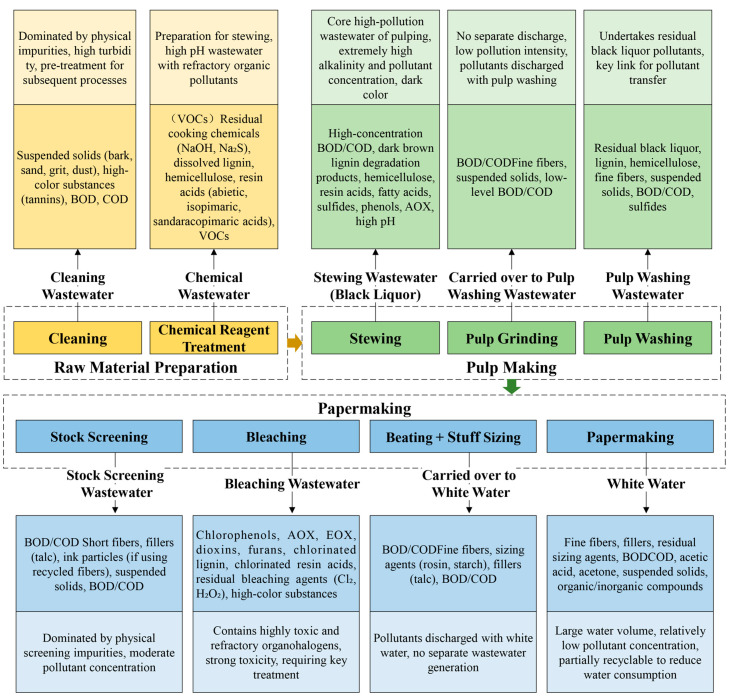
Released pollutants during the pulping and paper-making production process.

The papermaking process is the final stage in the pulping and paper-making industry. At this stage, bleached pulp is further processed to manufacture products such as printing paper, toilet paper, or tissues [45]. Its core stages include pulp preparation (beating, blending, and chemical addition), sheet forming, pressing, drying, and finishing. The wastewater generated in this stage is dominated by white water, which accounts for 70–90% of the total wastewater volume in the papermaking stage. Its characteristics include large water volume, low-concentration organic pollutants, high suspended solids content, and complex composition [8]. These characteristics are significantly affected by pulp type, paper grade, and process configuration. Newsprint and cultural paper production use more mechanical pulp, and the generated wastewater has lower COD (500–1000 mg/L) and higher fiber content. Packaging paper (corrugated board) production uses more fillers, leading to higher SS concentration (1000–2000 mg/L) in wastewater [46]. Recycled paper papermaking wastewater contains more stickies, ink residues, and microplastics, with a more complex composition and poorer biodegradability (BOD/COD ratio 0.2–0.4) [6,44]. Figure 3 systematically illustrates the categories, characteristics, and environmental implications of pollutants released throughout the entire pulping and paper-making production process, encompassing raw material preparation, pulping, and papermaking stages.

**Table 1:** Potential water pollutants from pulping and paper-making processes.

Process Stage	Key Process Step	Main Wastewater/Pollutant Source	Key Pollutant Types & Characteristics	pH	COD Concentration (mg/L)	BOD/COD Ratio	Pollutant Load Intensity	TSS (mg/L)	Biodegradability	Refs.
Raw Material Prep.	Debarking, chipping, washing	Wood/non-wood surface impurities, silt, extractives	Suspended Solids (SS), resin acids, fatty acids, tannins (color), pesticide residues, silicates (agro-based)	6.0–7.5	1000–3000	0.3–0.5	Low	7150	Good	[33,47,48]
Pulping	Chemical (Kraft)	BL, methanol, ethanol, furfural, reduced sulfur compounds (H_2_S, CH_3_SH)	Alkali lignin, lignin fragments, hemicellulose deg. products (formic, acetic acids), resins, fatty acids, residual inorganics (NaOH, Na_2_S); Volatile organic compounds (VOCs), Total reduced sulfur (TRS)	9–12	10,000–100,000	0.15–0.3	Very High (>90% of pulping load)	40	Poor	[19,47,48,49]
	Chemical (Sulfite)	Spent sulfite liquor (SSL)	Lignosulfonates, carbohydrates, sulfites, phenols, furan derivatives	1–3	5000–50,000	0.2–0.4	High (60–80% of kraft load)	-	Moderate	[27,50]
	Mechanical	Washing wastewater	Fiber fines, lignin fragments, resin acids, fatty acids, residual peroxides	4.0–7.5	1000–3000	0.3–0.4	Low (1/5–1/3 of chemical pulping)	330–510	Good	[31,33,48]
	Semi-chemical	Spent cooking/washing water	Partially dissolved lignin, DCS, residual Na_2_SO_3_, trace AOX	7.0–8.5	5000–15,000	0.25–0.45	Moderate (1.2–1.8× mech.; 40–60% of chem.)	-	Moderate	[24,25,33,37,38]
Bleaching	ECF/TCF Bleaching	Bleach plant effluent	AOX, chlorides, residual oxidants, lignin oxidation products, high color	2–12	2000–8000	0.15–0.3	Medium-High (high toxicity)	950	Poor	[39,41,44,51]
Papermaking	Stock prep. forming, pressing, drying	Whitewater, spills, dryer condensate	Fibers, fillers (clay, CaCO_3_), additives (starch, dyes, sizing), trace VOCs, microplastics	6.5–8.0	500–1500	0.25–0.4	Low-Medium	1241	Moderate	[6,44,46,49,51]

### 2.4. Key Factors Affecting Wastewater Characteristics and Pollutant Differences

#### 2.4.1. Raw Material Characteristics

The characteristics of P&P wastewater, as well as the differences in pollutant composition, concentration, toxicity and biodegradability of the effluent, are comprehensively influenced by raw material properties and process technology configuration. Clarifying these interrelated factors is crucial for optimizing process operations, reducing pollutant emissions, and formulating targeted wastewater treatment strategies, thereby promoting clean production in the P&P industry [52].

Raw material characteristics are the fundamental factor affecting wastewater properties, as lignocellulosic raw materials (e.g., wood, non-wood, and recycled paper) differ significantly in chemical composition and extractive content. Wood raw materials exhibit obvious differences between softwood and hardwood: softwood has a higher lignin content (25–35%)and is rich in extractives such as resin acids and terpenes [53], while hardwood has a lower lignin content with extractives dominated by fatty acids and phenols, leading to variations in the type and concentration of organic pollutants in the resulting wastewater [54,55]. This discrepancy results in the COD emission of BL from softwood kraft pulping being 30–50% higher than that from straw pulping [56].

Non-wood raw materials (e.g., wheat straw, rice straw, bamboo) are characterized by higher hemicellulose content (25–30%) and ash content, which not only increases the COD and SS of wastewater but also introduces specific pollutants such as silica (derived from straw), which may cause scaling problems in wastewater treatment systems [57]. Moreover, to mitigate reliance on timber resources associated environmental pressures, the application of non-wood raw materials (e.g., agricultural residues) has gained increasing attention. These raw materials possess high cellulose content and short growth cycles, exhibiting favorable sustainability and economic feasibility, and have become important pulping raw materials in some Asian countries, such as China and India, which heavily rely on bagasse and wheat straw, which together account for about 70% of the raw materials needed by the pulp industry [16]. However, their high ash content, high silicon content, and more complex impurity composition result in elevated inorganic salt concentrations and reduced treatability in pulping wastewater, imposing higher requirements on bleaching and wastewater treatment processes. Therefore, while promoting the diversification and sustainable use of raw materials, it is imperative to systematically assess the pollutant generation characteristics of different raw materials and optimize pulping processes and wastewater treatment systems accordingly, so as to achieve clean production and green development in the paper industry.

Recycled paper, as a secondary raw material, introduces additional pollutants including adhesives, ink residues, microplastics, and trace heavy metals (e.g., lead, cadmium) from additives, further increasing the complexity of wastewater composition and treatment difficulty [58].

#### 2.4.2. Processing Technologies

Process technology configuration is a key factor shaping wastewater pollution characteristics, with significant variations in pollutant emission intensity among different process routes. In terms of pulping processes, chemical pulping (kraft and sulfite processes) generates high-strength wastewater with high COD and poor biodegradability due to extensive lignin degradation and heavy chemical reagent usage. In contrast, mechanical pulping relies on physical shear and friction forces for fiber separation, involving minimal chemical input and thus producing low-pollution effluent [59]. Specifically, the BL pollution load of the kraft process is 1.5–2 times that of the sulfite process [60].

For agricultural waste pulping, the dosage of sodium hydroxide (6–14% of raw material weight) and high-temperature/pressure cooking conditions (150–160 °C, 6–7 atm) directly regulate the concentration of alkali lignin, hemicellulose degradation products, and organic loads (COD, BOD) in BL [36,61]. Post-cooking washing and screening processes generate wastewater with a solids content of 7–10%, containing residual alkali and soluble organic matter. Digester and evaporator condensates, though accounting for only 5% of the total mill wastewater volume, contribute 5–10% and 10–20% of the total plant BOD emissions in bleached and unbleached kraft pulp production, respectively [62].

Bleaching processes and water reuse systems further alter wastewater quality. Traditional chlorine-containing bleaching produces highly toxic wastewater with AOX concentrations of 500–2000 mg/L, while ECF bleaching reduces AOX emissions by 80–90% while maintaining pulp quality, making it the mainstream industrial technology. TCF bleaching achieves zero AOX emissions but requires higher agent dosage and stricter process control [63]. In papermaking, open-loop water systems lead to high wastewater discharge and fiber loss, whereas closed-loop white water recycling systems reduce discharge volume by 60–70% but may cause the accumulation of DCS, requiring targeted treatment to avoid process interference. Promoting clean bleaching technologies and optimizing process integration are critical to achieving green production [10].

#### 2.4.3. Operating Parameters and Process Control Levels

Process operating parameters and control precision further regulate the composition and concentration of pollutants, playing a decisive role in pollution reduction. In the chemical pulping of agricultural waste, cooking temperature (150–160 °C), pressure (6–7 atm), duration (approximately 6 h), and NaOH dosage (6–14% of raw material) directly determine the degradation degree of lignin and hemicellulose—higher alkali dosage and cooking intensity typically increase wastewater COD and chroma [64].

In the washing and screening stages, water allocation, reuse ratio, and washing duration (e.g., 4–6 h) not only affect fiber recovery rate but also determine the concentration of TSS and residual lignin in the washing wastewater [65]. In the bleaching process, the addition method of bleaching agents (e.g., two-step addition of calcium hypochlorite), reaction residence time (approximately 2 h), and post-bleaching wash water quality control are key factors influencing AOX and residual chlorine content; improper control may lead to excessive accumulation of persistent organic pollutants such as chlorinated lignin [66].

During papermaking, operating parameters including wire section spray water usage, white water reuse rate, and steam consumption in pressing and drying stages also significantly impact wastewater discharge and pollutant load. Thus, scientific management of operating parameters and improvement of process control precision are essential for realizing clean production in the pulping and paper-making industry [67].

## 3. Common Treatment Methods for Pulping and Paper-Making Wastewater

P&P wastewater is characterized by high organic load, intense coloration, and complex pollutant composition. In response to these challenges, a variety of treatment technologies have been developed, which can be broadly categorized into physical, chemical, biological, electrochemical, and integrated treatment methods. Each method presents distinct advantages and limitations, which are closely dependent on the characteristics of P&P wastewater and specific treatment objectives. Table 2 compares their major target pollutants, representative treatment performance, principal advantages, key limitations, applicable wastewater scenarios, and engineering maturity.

### 3.1. Physical Treatments

As a fundamental primary pretreatment technology for P&P wastewater, physical treatment achieves pollutant separation by exploiting differences in physical properties (e.g., density, particle size, surface properties) between pollutants and water, without altering the chemical structure of contaminants. It is primarily utilized to remove suspended solids, fiber fines, and partial colloidal substances, reduce the pollutant load of subsequent treatment units, and lay a critical foundation for the efficient operation of subsequent chemical or biological treatment processes. Compared with other treatment methods, physical treatment processes offer advantages, including simple equipment configuration, low energy consumption, rapid treatment efficiency, and no secondary pollution caused by chemical reagents, making them an indispensable component in P&P wastewater treatment systems.

Common physical treatment methods for P&P wastewater include sedimentation, flocculation precipitation, filtration, centrifugation, flotation, and membrane filtration [71]. Sedimentation is a simple and cost-effective approach for removing suspended solids from wastewater, relying on gravity to achieve the solid–liquid separation of suspended matter. The sedimentation efficiency can be effectively improved by adding flocculants to the wastewater system. 

Flocculation precipitation removes suspended fine particles, colloidal macromolecules, and micro-pollutants from water through the formation of flocculent aggregates by coagulants via multiple mechanisms. This process can effectively reduce key pollutants in P&P wastewater, including COD, AOX, chroma, and lignin components that mostly exist in the form of 500 nm colloids in wastewater. The selection of coagulants is crucial for the treatment efficiency, as their surface charge and chemical properties directly determine the interaction efficiency with target pollutants. For instance, lignin particles are typically negatively charged in the aqueous phase; thus, cationic polymeric coagulants can neutralize the negative charge of lignin particles, promoting their aggregation and separation from water. pH control is also a key factor affecting the treatment efficacy: traditional inorganic coagulants such as alum (Al_2_(SO_4_)_3_) achieve the optimal lignin recovery efficiency within the pH range of 2–7 based on the charge neutralization mechanism [72]. A mixed precipitation-coagulation system has been reported to achieve 84% COD removal and 89% decolorization after primary sedimentation combined with magnesium sulfate addition [73]. Alum, ferric chloride (FeCl_3_), and polyaluminum chloride (PAC) are the most commonly used inorganic coagulants for P&P wastewater treatment [74]. Under acidic conditions, polymeric coagulants such as polyethylene oxide (PEO), PAC, and polydimethyldiallylammonium chloride (PDDM) can be applied to BL treatment, achieving a COD removal rate of 50–70% [75]. Flocculation precipitation can remove most suspended solids from P&P wastewater to realize primary water purification and fiber recovery; its selection and application strategy are highly dependent on papermaking raw materials, production processes, and actual pollutant composition of wastewater [76]. Research by Ekstrand et al. indicated that for P&P wastewater with extremely high cellulose content, direct anaerobic digestion without primary sedimentation can produce more biogas compared with the traditional process route of “sedimentation-dewatering-incineration”, suggesting that pursuing the ultimate solid–liquid separation is not the optimal solution in certain specific scenarios [77]. In recent years, the potential environmental and health risks associated with traditional coagulants such as alum and FeCl_3_ have attracted widespread attention, which has driven the research and application of natural alternative coagulants. Chitosan, moringa seed powder, and plant-based mucilage have been recognized as low-toxic and high-efficiency alternative coagulants for P&P wastewater treatment due to their excellent flocculation performance and environmental benignity.

Filtration is another key physical treatment method that uses filter media (e.g., sand, gravel, filter cloth, activated carbon, and membrane materials) to separate suspended solids from wastewater, and the filtration efficiency can be further improved by the pre-addition of flocculants or coagulants [78].

Centrifugation utilizes centrifugal force generated by high-speed rotation to achieve rapid separation of suspended solids from water, while flotation realizes the solid–liquid separation by making air bubbles absorb suspended solids in wastewater, forming solid-bubble aggregates that float to the water surface for collection and removal. Membrane filtration achieves pollutant separation by using porous membrane materials as the separation medium. Although the aforementioned physical treatment methods can effectively remove suspended solids and partial organic matter from P&P wastewater, their removal performance against toxic, refractory small molecule pollutants is relatively limited, making them unable to independently meet full-standard discharge requirements.

Membrane technology, which can not only separate solids from water but also realize the efficient separation of solutes and solvents, is regarded as a promising sustainable treatment technology. It can completely or partially replace the traditional energy-intensive and high-pollution separation processes, thus reducing the operational cost of industrial wastewater treatment and improving the environmental performance of the process. Valderrama et al. investigated the feasibility of membrane filtration for the treatment of P&P wastewater BL, and the results showed that membrane treatment could effectively remove organic compounds and inorganic salts from BL, with the removal rates of total organic carbon (TOC), CO_3_^2−^, sulfate, Na^+^, and Mg^2+^ reaching 92.5%, 84.10%, 88.7%, 73.21%, and 99.99% on average, respectively [79]. Tonni et al. studied the application of reverse osmosis (RO), a typical membrane filtration technology, in the treatment of P&P process whitewater, and found that RO could achieve almost complete removal of SiO_2_ from whitewater under an operating pressure of 10 bar, indicating that RO-treated whitewater can be directly reused without further treatment. In view of the application of membrane technology in P&P wastewater treatment, many researchers have carried out in-depth studies on membrane combined treatment processes. Nanofiltration (NF) technology combined with ultrafiltration (UF) pretreatment has been developed, and an integrated treatment process consisting of pretreatment, ultrafiltration, and reverse osmosis has been further constructed to evaluate its treatment effect and practical application value. The research results showed that the combination of NF and UF pretreatment can effectively reduce the operating load of traditional recovery boilers; meanwhile, the integrated pretreatment UF-RO process can significantly improve the quality of recycled wastewater, ensuring that the treated effluent meets the relevant quality standards for industrial production water, thus providing a feasible technical approach for the resource utilization and recycling of P&P wastewater [80].

In summary, physical treatment methods exhibit high treatment efficiency and good removal effect on suspended solids and partial colloidal pollutants in P&P wastewater. However, these methods also have inherent limitations, such as the generation of large quantities of sludge waste during the treatment process and relatively high operational costs for some advanced physical technologies (e.g., membrane filtration). Accordingly, physical units are generally adopted as essential pretreatment or polishing units rather than independent treatment systems for complex P&P effluents.

### 3.2. Biological Treatments

Biological treatment serves as the core process for P&P wastewater purification, leveraging the metabolic activities of microorganisms to decompose organic pollutants into harmless substances, including water, carbon dioxide and microbial biomass. This method can be operated as an independent treatment unit or integrated with physical and/or chemical processes to form a combined treatment system, thus adapting to different pollutant loads and treatment requirements [81]. Aerobic treatment processes (e.g., activated sludge process and aeration tank process) are the mainstream technologies for P&P wastewater treatment. These processes depend on aerobic microorganisms (e.g., bacteria, fungi) to oxidize and decompose organic pollutants under aerobic conditions, achieving efficient pollutant removal. They yield an extremely high BOD removal rate (>95%) and produce high-quality effluent with low pollutant concentrations, making them particularly suitable for treating low-to-medium concentration P&P wastewater. However, their COD removal capacity is relatively limited, generally ranging from 40% to 60%, especially for refractory organic compounds (e.g., lignin derivatives) that are difficult to biodegrade by aerobic microorganisms. In addition, aerobic treatment processes are usually accompanied by high energy consumption and excess sludge production, which increases the subsequent cost of sludge treatment [82]. Thus, despite the reliable effluent quality achieved by aerobic processes, their practical application is restricted by high operating costs [83]. Anaerobic treatment processes, by contrast, decompose organic pollutants under anoxic conditions through the metabolic activity of anaerobic microorganisms, and the simultaneous production of biogas (mainly methane and carbon dioxide) enables resource recovery from P&P wastewater. It should be noted that anaerobic microorganisms are highly sensitive to toxic compounds (e.g., organochlorine compounds, resin acids); therefore, bleaching sulfate pulp wastewater containing such toxic substances is not suitable for direct anaerobic treatment. Although anaerobic processes exhibit significant potential in resource recovery (biogas utilization) and operating cost reduction, they have poor adaptability to fluctuations in wastewater quality, and the effluent quality after anaerobic treatment usually fails to meet direct discharge standards [33]. Conventional biological systems are easily inhibited by toxic and recalcitrant components in P&P wastewater, necessitating auxiliary advanced treatment strategies. Emerging advanced oxidation and electrochemical approaches are generally applied as targeted pretreatment to break down biotoxic macromolecules, reduce microbial inhibition, and improve the overall biodegradability of raw wastewater. In practical applications, aerobic and anaerobic processes are often optimized or combined (e.g., anaerobic-aerobic combined process) according to the actual concentration, toxicity of P&P wastewater and specific treatment objectives.

### 3.3. Chemical Treatments

Chemical treatment is one of the core technical approaches for treating complex P&P wastewater containing refractory pollutants (e.g., lignin, AOX, persistent COD). Traditional chemical treatment processes rely on chemical reactions, mainly including coagulation-precipitation, conventional oxidation and adsorption. These methods remove pollutants by adding appropriate chemical reagents and utilizing specific reaction mechanisms, with the advantages of mature technology, simple operation, high treatment efficiency and recyclable adsorbents. Eskelinen et al. achieved up to 90% removal of COD by using chemical precipitation with 5 g/L CaO, demonstrating its effectiveness [84].

Among advanced oxidation processes (AOPs), ozonation has been applied to convert refractory compounds into less harmful substances. Ozonation may reduce toxicity and improve the degradability of chlorine-based compounds during treatment [85]. However, it remains economically challenging and may interfere with downstream biological treatment [86]. Moreover, secondary reactions of ozone with primary reaction by-products may generate new toxic compounds, such as ketones, aldehydes, and organic acids. To address this issue, Fenton-like oxidation (Fe^3+^/H_2_O_2_) can be employed. In this process, H_2_O_2_ reacts with Fe^3+^ in solution to generate hydroxyl radicals(•OH), which attack organic chemicals by virtue of their high energy. The short lifespan of these hydroxyl radicals, along with their selectivity toward the pollutants, is regarded as an advantage of this process. Oxidant concentration, temperature, treatment time and pH are critical parameters in the Fenton-based process. In particular, a pH of 2.5–3.0 is optimal for obtaining higher COD and color removal [87]. However, limited lignin solubilization and hydroxide precipitation under acidic conditions impede Fenton-based processes [88]. Compared with the conventional Fenton process, the photo-Fenton process exhibits higher organic removal rates [89]. Nevertheless, as a light-dependent system, the photo-Fenton process is inherently restricted by light availability and is not yet directly applicable for uninterrupted 24/7 continuous industrial wastewater treatment under natural operational conditions.

AOPs exhibit remarkable treatment effects: they can effectively degrade pollutants that are difficult to remove by traditional methods and decompose macromolecular organics into small-molecule substances that are easily biodegradable by microorganisms in subsequent processes. To optimize the overall treatment effect, combined processes have been investigated. The combination of AOPs and biological treatment can exert synergistic advantages, significantly improving the overall treatment efficiency and further increasing the COD removal rate by approximately 20%, thus meeting the industrial requirements for zero discharge and water reuse [90]. Nevertheless, the practical application of chemical oxidation still faces prominent challenges, such as strict pH dependence, high reagent consumption, immature large-scale application technology, and high operational costs [4].

### 3.4. Electrochemical Treatments

Electrochemical methods for wastewater treatment have attracted increasing interest due to their outstanding capacity to eliminate various pollutants. In the treatment of P&P wastewater, electrocoagulation and electrochemical oxidation have been extensively investigated as alternative or supplementary purification strategies.

#### 3.4.1. Electrocoagulation Technologies

Electrocoagulation (EC) has emerged as a highly efficient and sustainable electrochemical strategy for the remediation of pulp and paper wastewater, outperforming conventional chemical coagulation–flocculation–sedimentation (CCFS) in multiple aspects. Extensive studies have validated its applicability: Zaied et al. achieved 98% COD, 92% polyphenols and 99% color removal from paper BL using aluminum-electrode EC under optimized conditions (initial pH 7, 50 min, 14 mA·cm^−2^) [91]; Buzzini et al. confirmed that EC removed 67% residual COD without coagulant addition, while CCFS required 350–400 mg·L^−1^ aluminum sulfate and cationic polymer for 89% residual COD removal [92]; Pandey et al. realized over 80% removal of COD, TOC, TDS and color via continuous-flow aluminum-electrode EC (pH 5.0, 10.72 mA·cm^−2^, 0.1 L/min, 120 min) [93]; and Dogan et al. further developed a hybrid EC/electrooxidation-oxone process that attained 98.42% COD, 98.61% color and 99.4% tannin/lignin removal [94]. Relative to CCFS, EC generates coagulants in situ via sacrificial electrode dissolution without external chemical dosing, reduces sludge production and secondary pollution, integrates flocculation and electroflotation for enhanced pollutant trapping, and enables automatic pH neutralization alongside flexible batch, continuous or hybrid operation. However, the large-scale industrial implementation of EC is still restricted by several critical limitations: sacrificial Al/Fe electrodes suffer from rapid consumption, surface passivation and short service life, elevating long-term operational costs; high current densities lead to relatively high energy consumption and poor economic viability; single EC processes fail to fully mineralize refractory lignin and polyphenolic compounds; and the scale-up of continuous-flow EC reactors with optimized hydrodynamics and electrode configuration lacks sufficient engineering validation.

#### 3.4.2. Electro-Oxidation Technologies

Electrocatalytic oxidation (ECO) is an advanced wastewater treatment technology that degrades organic pollutants via redox reactions on the electrode surface. Driven by electron transfer, it promotes the formation of reactive intermediates and may convert pollutants into smaller, less harmful compounds, and in some cases into mineralization products such as CO_2_ and H_2_O. Its main advantages include high oxidation efficiency, operational tunability, and suitability for refractory pollutants.

An ECO system comprises an external power supply, electrolyte, electrodes, and electrocatalyst, among which the electrocatalyst/electrode material is the core factor determining the treatment efficiency. The electrolyte (e.g., organic/inorganic acids and bases, molten salts, ionic liquids) ensures high ionic conductivity and mass transfer efficiency. Electrodes are fabricated from conductive materials, including base metals (Ni, Co, Cu), modified precious metals, and carbon-based materials. The electrocatalyst—either the electrode material itself or its surface coating—reduces electrolysis overpotential, thereby improving degradation efficiency.

**Figure 4:**
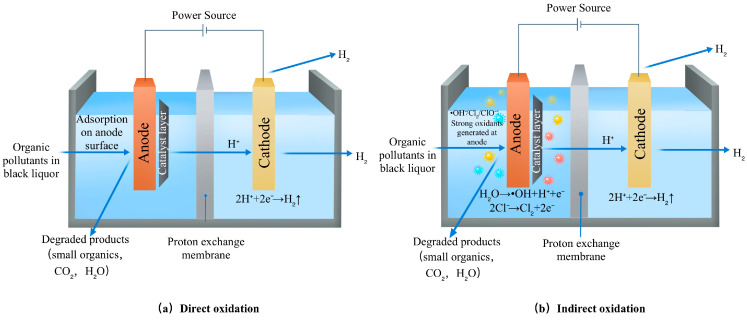
Mechanism of the ECO process.

The electrochemical degradation of organic pollutants in P&P wastewater proceeds via two synergistic mechanisms (depicted in Figure 4): (a) Direct Oxidation: Pollutants are adsorbed onto the anode surface and then degraded via direct electron transfer; (b) Indirect Oxidation: Degradation occurs in the bulk solution, mediated by electrochemically generated strong oxidants (e.g., hydroxyl radicals (•OH), chlorine, hypochlorite, ozone, hydrogen peroxide), which can effectively attack the macromolecular structure of lignin and break its chemical bonds [95].

Lignin in BL is oxidized at the anode with the assistance of a catalyst layer driven by an external power supply, producing biodegradable and environmentally benign oxidation products, such as low-molecular-weight organic acids and aromatic monomers [96]. H^+^ ions pass through the proton exchange membrane and are reduced at the cathode to produce H_2_, realizing resource recovery. This clearly reflects the dual mechanism of direct anodic oxidation + indirect oxidation and the core role of transition metal-based catalysts.

The performance of ECO systems is strongly determined by electrode and catalyst materials. Precious-metal-based catalysts exhibit high activity but are limited by high costs and poor accessibility for industry promotion, whereas transition-metal-based catalysts (Mn, Fe, Co, Ni, Cu) have attracted greater attention due to their lower cost and promising performance in near-neutral or alkaline media relevant to many P&P wastewater streams. Zirbes et al. reported that lignin-derived compounds could be selectively converted into products such as vanillin [97]. Smith et al. showed that nickel-based anodes could promote lignosulfonate depolymerization with vanillin yields of 5–9.6% (*w*/*w*) [98]. Lower current density and lower substrate concentration were found to favor product formation, whereas higher current density tended to promote vanillin overoxidation. Yan et al. also reported that nickel-foam-derived electrodes enabled lignin depolymerization under alkaline conditions, with the combined yield of vanillin and syringaldehyde reaching 17.5% [99]. These studies demonstrate promising laboratory-scale performance and indicate the potential of transition metal-based catalysts for further development in lignin valorization. However, Ndambakuwa et al. highlighted that catalyst durability in sulfide-containing P&P wastewater remains a major limitation, as catalytic activity and service life often exhibit a trade-off in complex wastewater matrices [100].

Transition metal oxides and transition metal salts represent two additional catalyst classes. Xu et al. reported that a Cu^I^/Cu^II^@BaTiO_3_ heterointerface enhanced charge transfer and radical formation in P&P wastewater [101]. Ma et al. showed that a spinel CuxCo_1_−xMn_2_O_4_ electrode retained 84.9% of its catalytic efficiency after five cycles, indicating encouraging laboratory-scale stability [102]. Bjørsvik et al. used cobalt salts for oxygen-assisted lignin oxidation in an alkaline system, achieving vanillin yields of 4–7% [103]. Yang et al. conducted research using a proton exchange membrane electrolysis cell, where the Fe^3+^/Fe^2+^ redox pair served as a highly efficient catalyst, enabling low-energy electrocatalytic conversion of biomass (including cellulose, lignin, etc.). This system operate at a low cell voltage of about 0.7 V, and the entire process consumes only 1.845 kW·Nm^−3^ H_2_, saving about 60.74% of energy compared to traditional processes [104]. Shen et al. further highlighted that transition-metal-based catalytic systems remain highly important in lignin upgrading because their tunable redox properties and surface active sites can promote bond cleavage and product-oriented conversion, thereby providing an important basis for the further development of transition-metal-oxide electrocatalysts [105]. ECO can achieve the harmless degradation and lower energy consumption, energy, offering a feasible supplementary solution for the resource-based treatment of P&P wastewater.

Electrochemical advanced oxidation processes (EAOPs), which use electrons as clean reactants, mineralize organic pollutants via in situ generation of strong oxidants (primarily •OH). Importantly, EAOPs are not universally applicable for bulk P&P wastewater; instead, they are most suitable for specific scenarios, such as pretreatment to enhance biodegradability, treatment of refractory or toxic side-streams, or advanced treatment steps in integrated systems. Research on integrating electrocatalysis with Electro-Fenton technology for P&P wastewater treatment has achieved notable progress. Yang et al. constructed the Electro-Oxidation-Persulfate-Electro-Fenton (EO-PS-EF) tri-coupling system, which exemplifies this strategy. Through the synergistic activation of persulfate (PS) by the boron-doped diamond (BDD) anode and graphite felt (GF) cathode, this system efficiently generates hydroxyl radicals (•OH) and sulfate radicals (SO_4_•^−^) via dual catalytic cycles, markedly enhancing the degradation efficiency of refractory organic pollutants and system stability [106]. Ozonation, another highly effective AOP, has been widely reported for efficient decolorization of P&P wastewater, Ozone rapidly generates •OH that reacts with organic pollutants, and coupling ECO with ozonation promotes both ozone generation and decomposition, thereby enhancing overall oxidation efficiency. Gu et al. developed an electrocatalytic ozone system where applying a current through stainless steel-graphite electrodes accelerated ozone decomposition to generate singlet oxygen (^1^O_2_), enabling efficient organic degradation with a reaction rate constant (0.387 min^−1^) four times higher than that of ozonation alone, without metal ion leakage to avoid secondary pollution [107]. Du et al., in Water Research, developed an integrated system of nanofiltration-electrocatalytic membrane reactor-ozone oxidation. This system uses a conductive carbon membrane as the cathode and graphite as the anode to construct an electrocatalytic membrane reactor (ECMR), which is then coupled with ozone to treat nanofiltration concentrate from textile dyeing wastewater. An electric field enhances the decomposition of ozone at the cathode, generating a large amount of •OH. Simultaneously, the high concentration of sodium sulfate in the nanofiltration concentrate provides a highly conductive environment for the electrocatalytic reaction. The porous structure of the conductive carbon membrane also improves the mass transfer efficiency of ozone. This coupled system achieves a COD removal rate of 92.7% for the nanofiltration concentrate, far exceeding that of ozone oxidation alone (57.7%) and the electrocatalytic membrane reactor alone (13.9%). The final effluent COD is reduced to 48.6 mg/L, meeting the standards for recycled water in textile dyeing and printing [108]. It also achieves efficient recovery and reuse of sodium sulfate, exhibiting favorable economic and environmental benefits. Regarding technological maturity, most EAOP systems remain at the laboratory or pilot scale; scale-up challenges include high energy consumption, high cost of electrode materials, and electrode fouling, which restrict continuous industrial operation.

#### 3.4.3. Electroreduction Technologies

As a complementary technology to ECO, electroreduction technology has unique application value in P&P wastewater treatment. Compared with anodic oxidation, research on lignin conversion via cathodic electroreduction is relatively limited [109]. Electrocatalytic hydrogenolysis is one of the most promising pathways for the selective depolymerization of lignin in P&P wastewater [109,110], enabling the selective generation of target products with low energy consumption under mild conditions through precise control of electrode voltage. Related studies have confirmed its outstanding advantages in selectivity, energy consumption, and resource recovery potential. The hydrogenation products are mainly aromatic compounds, saturated cyclic alcohols, and phenolic derivatives, and the product distribution is closely related to the catalyst type and reaction conditions. Electro-reduction technology provides an efficient and controllable pathway for the high-value conversion of lignin in P&P waste liquor. Under the catalysis of different cathode materials such as FeNiCr_18_(304), FeW_9_Cr_4_V_5_Co_3_ alloy, copper cathode and Raney nickel, lignin can be directionally converted into G-type compounds (guaiacol, vanillin, etc.), S-type compounds, phenols, high-value aromatic aldehydes and organic acids [111]. The 4-O-5 type lignin dimer can be converted into cyclohexanol through hydrogenolysis-hydrogenation synergistic reaction [112], while the β-O-4 type lignin model compound will generate a mixture of phenols and cyclohexanol [113]. In specific systems, deep hydrogenation and deoxygenation can also be achieved to obtain aromatic products. These products cover the fields of fine chemicals and biofuel precursors, providing diversified pathways for the resource utilization of P&P waste liquor [114].

In practical application, electroreduction technology achieves the targeted conversion of P&P wastewater pollutants through cathode electron transfer or electrogenerated reducing agents, further confirming its specific application orientation. Taking pulp whitening as an example, with anthraquinone-2-sulfonic acid (AQS) as the redox mediator, hydrogen peroxide is in situ generated at the carbon cloth cathode for pulp whitening, which increases the International Organization for Standardization (ISO) brightness of thermomechanical pulp by 21% and reduces yellowness by 15%. However, its industrial application is limited by electrode scaling caused by AQS crystallization [115]. In terms of resource recovery, the electrolysis of P&P wastewater BL can realize the integrated goal of pollution control-resource recovery: the cathode chamber produces hydrogen and sodium hydroxide (with a sodium recovery rate of 80.4%), and the anode recovers lignin and other biomass solids (with a recovery rate of 76%) [116]. In addition, the cathode-coupled Fenton/Fenton-like reaction can degrade persistent organic pollutants in P&P wastewater through the Fe^2+^/Fe^3+^ cycle, but excess Fe^2+^ will cause free radical quenching and reduce treatment efficiency [117]. Electroreduction technology is still in the laboratory research stage; scale-up challenges include high catalyst costs (e.g., Raney nickel), poor catalyst durability in complex wastewater matrices, and difficulty in separating low-concentration target products, which hinder continuous industrial operation.

#### 3.4.4. Integration of Electrocatalytic Oxidation with Biotechnologies

Although electrochemical technologies and AOPs can achieve high COD removal efficiencies ranging from 60% to 100%, their practical application is restricted by high operational cost, mainly due to considerable energy consumption (up to 96 kWh/kg COD removed) [118]. By contrast, biological treatment represents a sustainable alternative that complies with strict environmental regulations and generates minimal secondary pollution. Microbial degradation is widely applied in wastewater treatment owing to its high efficiency, low energy demand, and good adaptability, enabling the conversion of complex organic pollutants into harmless small-molecule products. Nevertheless, conventional biological processes alone show limited performance toward refractory contaminants (e.g., lignin and aromatic compounds) and are highly sensitive to operational conditions such as temperature, pH, and dissolved oxygen, creating a clear niche for electrocatalytic pretreatment to enhance biodegradability.

Thus, bio-electrochemical systems (BESs), which integrate biological and electrochemical processes via redox reactions, have attracted significant attention as a promising strategy for environmental remediation [119]. Hybrid systems combining ECO with biotechnology can overcome the limitations of individual technologies and achieve synergistic treatment effects. ECO pretreatment effectively degrades macromolecular organic pollutants, thereby improving biodegradability of wastewater and facilitating subsequent microbial decomposition. The subsequent biological stage further removes the refractory intermediates formed during electrocatalytic oxidation, reducing the electrocatalyst load and improving overall system efficiency.

As a typical BES configuration, MFC enable simultaneously wastewater treatment and energy recovery by converting chemical energy stored in organic matter into electricity [120]. In the MFC system, microorganisms act as biocatalysts to drive electrochemical reactions. Saccharomyces cerevisiae, Proteus vulgaris, and anaerobic bacteria extracted from sludge or wastewater in the anode chamber can directly oxidize the organic components in pulping wastewater through their life activities, extracting electrons from the fuel through respiration and fermentation. The fuel is ultimately oxidized to CO_2_, and the electrons are transferred to the cathode through an external circuit, where they combine with O_2_ to generate H_2_O under the catalysis of cathode catalysts such as Pt/C [121]. Adding redox mediators can further accelerate the transfer of electrons to the electrodes, further improving battery performance [122,123]. Logan’s research group at Pennsylvania State University was the first to apply MFC technology to P&P wastewater treatment [124]. Since then, related technologies have been continuously upgraded: Sathe et al. combined MFC with electro-Fenton oxidation to develop a BEF system. This system releases electrons and protons by oxidizing organic substrates with anodic electroactive microorganisms, and generates H_2_O_2_ in situ at the cathode to initiate the Fenton reaction. It can efficiently degrade emerging pollutants such as drugs and dyes without external energy input, and can achieve a COD removal rate of more than 90% with extremely low energy consumption [125]. In addition, Khater et al. reported that an MFC equipped with a NiO–CuO/G cathode electrocatalyst delivered a power density of 21.3 mW/m^2^ and enhanced oxygen reduction reaction (ORR) efficiency [126].

In contrast to MFCs, microbial electrolysis cells (MECs) require a small external bias to overcome the thermodynamic barrier of cathodic hydrogen evolution, thereby shifting the main product from electricity to biohydrogen. MECs are more efficient in treating high-concentration and refractory wastewater. Narasimman Mathimani et al. reported a hybrid MFC–MEC system in which photosynthetic bacteria converted organic components in wastewater under illuminated conditions, achieving nearly 80% COD removal together with hydrogen production [127]. It indicated that MEC-associated BES configurations may offer additional routes for wastewater treatment and resource recovery. 

Popat et al. demonstrated that Electro-Fenton (EF) pretreatment enhanced wastewater biodegradability. Under optimized conditions (10 V, catalyst 10 mg/L, persulfate 200 mg/L), the EAOP reduced the COD of mixed industrial wastewater from 1152 mg/L to 691 mg/L and increased the BOD/COD ratio from 0.34 to 0.52, greatly facilitating the subsequent biological process. Further biological treatment decreased the COD to 60.8 mg/L, giving an overall removal rate of 94% [128]. However, given that this persulphate-assisted system has only been demonstrated at laboratory batch scale, its direct application to mainstream treatment of very large P&P wastewater volumes would likely be difficult to justify in terms of oxidant demand, process continuity, and overall economics. Similarly, Aboudalle et al. investigated the combination of EF pretreatment with bioaugmentation for metronidazole wastewater treatment. By using acclimated Pseudomonas and Acetobacter strains, the mineralization efficiency increased from 58.1% (single biological treatment) to 97%, and the treatment time was shortened by 16 days [129]. This approach enhanced microbial tolerance to electrocatalytic byproducts via in situ acclimation, promoting the degradation of refractory organics. For P&P wastewater, a comparable strategy can be adopted: electrochemical oxidation is first employed to depolymerize lignin macromolecules, followed by a biofilm reactor or bioaugmented microbial system for efficient removal of high-concentration pollutants and resource recovery (Table 3).

## 4. Biomass Fuel Cells for Treatment and Valorization of Pulping Wastewater

**Figure 5:**
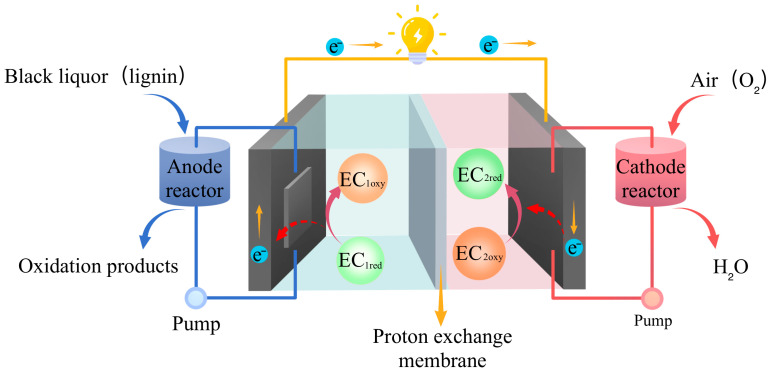
Schematic diagram of DBFCs.

Biomass fuel cells have emerged as an attractive research direction for P&P wastewater treatment, owing to their unique capability of integrated pollutant degradation and synchronous recovery of energy and value-added products. Among these systems, direct biomass fuel cells (DBFCs) (depicted in Table 4 and Figure 5) represent an important development pathway, which employ an asymmetric configuration consisting of an anolyte and catholyte. This design enables the direct utilization of alkaline pulping BL without elaborate pretreatment, achieving efficient pollutants (e.g., lignin) oxidation and simultaneous electricity generation.

The development of DBFC systems can be traced back to 1985, when Weetal et al. first reported a fuel cell system using anthraquinone-2-sulfonic acid as a liquid catalyst and sulfate lignin as fuel. However, the peak power density was only 0.34 mW/cm^2^ [135]. In 2014, Liu et al. proposed a DBFC based on phosphomolybdic acid as a liquid catalyst, increasing the peak power density to 0.55 mW/cm^2^ under solar irradiation with lignin as the feedstock [136]. In 2016, Zhao et al. further optimized the system by applying phosphomolybdic acid as a liquid catalyst for both anode and cathode, enabling a peak power density of approximately 5 mW/cm^2^ with sulfate lignin as fuel [137]. Zu et al. developed a low-temperature symmetric biomass flow fuel cell mediated by the Fe^3+^/Fe^2+^ redox couple, which achieved direct electricity conversion from biomass residues like sugarcane bagasse and rice straw without noble metal catalysts. Fe^3+^ at the anode oxidized biomass into high-value chemicals and was reduced to Fe^2+^, and the potential difference between anodic Fe^2+^ and cathodic Fe^3+^ drove power generation; the consumed cathodic Fe^3+^ was rapidly regenerated by the synergistic effect of O_2_ and HNO_3_. This Fe-based cell exhibited excellent performance, with a peak power density of 54.5 mW/cm^2^ for sugarcane bagasse, stable discharge at 200 mA/cm^2^ for over 7 h and the ability to power 43 LED bulbs with a 100 cm^2^ active area, providing a low-cost and high-efficiency technical route for biomass-to-electricity conversion [138]. Ouyang et al. further constructed an electron transport chain mediated by ferricyanide and vanadyl (V) redox couples with an acid-alkaline asymmetric design for direct lignin fuel cells, using corn stover alkaline lignin as the fuel, and achieved a record high peak power density of 200.3 mW/cm^2^ and an overall electron transfer efficiency of about 90%, demonstrating that rational redox mediator design systems could significantly improve DBFCs performance [139]. Nowadays, DBFC systems can utilize a wide variety of liquid catalysts, including polyoxometalates (e.g., phosphomolybdic acid, phosphovanadomolybdic acid, silicotungstic acid), organic redox mediators (e.g., anthraquinone-2-sulfonic acid, methylene blue), and inorganic redox couples (e.g., FeCl_3_, Fe(NO_3_)_3_, CuCl_2_). However, DBFC still faces a critical bottleneck: most existing systems rely on acidic electrolytes, which tend to induce lignin condensation and the formation of refractory precipitates. This phenomenon impedes efficient electron extraction from lignin and renders them incompatible with alkaline pulping black liquor. Therefore, the development of electrocatalytic systems adaptable to alkaline environments and capable of selectively cleaving lignin β-O-4 bonds has become a key focus for DBFC optimization. Besides that, even liquid catalytic systems offer flexibility in catalyst selection and may avoid some surface-poisoning problems associated with heterogeneous electrodes, but their practical application in large volume wastewater system still faces challenges. Catalyst recovery, long-term operational stability, system integration and overall process economics impedes the scaling-up applications.

Recently, Ouyang et al. developed a LFFC using CoS@Ni foam as a solid electrocatalyst. In this system, lignin undergoes depolymerization and deep oxidation via the oxidative cleavage of β-O-4′ aryl ether bonds, accompanied by partial mineralization to CO_2_. A peak power density of 176 mW/cm^2^ was achieved at 80 °C, with an extraordinarily high anodic selectivity of 97% for lignin oxidation, greatly enhancing the practical feasibility of the technology [140]. Subsequently, within the same LFFC system, Gao et al. proposed a novel pathway to produce high value-add products (new antioxidants) from lignin by using CuSO_4_ as the anodic catalyst. Based on this electrocatalytic lignin conversion framework, Gao et al. further investigated cobalt as anodic catalyst, achieving a maximum power density of 199.8 mW/cm^2^ and aromatic aldehydes yield of 7.8% at a lignin concentration of 0.5 g/L [141]. These studies suggest the potential of LFFC systems for coupled lignin conversion and energy recovery. However, for dilute bulk wastewater, the recovery of low-concentration aromatic products from dilute bulk P&P wastewater is economically unfeasible. Therefore, such systems are more suitable for lignin-rich side streams. For the DBFC system, the development of more efficient and sustainably recyclable catalysts, along with the integration of DBFCs with other physical and biological treatment technologies, is expected to facilitate the efficient treatment of pulp and paper wastewater in the future.

## 5. Conclusions

P&P wastewater remains a highly challenging wastewater matrix because of its high organic load, strong color, toxicity, and the presence of refractory pollutants such as lignin and AOX. Existing physical, chemical, biological, electrochemical, and integrated treatment technologies each possess distinct advantages. Overall, the studies reviewed here indicate that coupled treatment strategies are particularly valuable, as they can combine complementary mechanisms and improve the removal of refractory pollutants and improve wastewater biodegradability. In this sense, integrated processes are more suitable than single-unit treatments for handling complex and compositionally variable P&P effluents. In particular, electrocatalytic treatments, including electrochemical advanced oxidation processes and electroreduction, exhibit significant potential for promoting refractory pollutants conversion and enabling the coupling of treatment and resource recovery, as exemplified by direct biofuel cells (DBFCs). It is important to note that electrocatalytic methods remain at the laboratory scale, constrained by high costs and large-scale catalyst preparation. Rather than replacing mature technologies (e.g., biological processes), electrochemical oxidation serves as a critical “cutting-edge reserve technology” to address unsolvable challenges of current methods. Its core objectives include deep removal of biologically recalcitrant organics (e.g., lignin derivatives), efficient decolorization and detoxification for stricter discharge standards or water reuse requirements, exploration of green reagent-free technologies to reduce secondary pollution, and precise oxidation control for converting pollutants into high-value-added chemicals or energy, thereby facilitating wastewater resource recovery and supporting the P&P industry in meeting future higher environmental requirements and “zero discharge” goals.

For the large-scale industrial application of electrocatalytic and biomass fuel cell technologies, key determinants of technical and economic feasibility include electrode area demand, electrolyte impurity tolerance, reagent consumption, catalyst or mediator recovery, and low-concentration product separation. Accordingly, future research should focus on the following priorities: (1) developing robust, impurity-tolerant catalyst systems to address durability issues in complex P&P wastewater matrices; (2) implementing source separation and targeted treatment of lignin-rich side-streams, which represent the optimal application niche for these technologies; (3) improving the integration between electrochemical and biological units to enhance synergistic effects and reduce operational costs; (4) deepening the understanding of lignin transformation pathways to optimize product selectivity and pollutant removal efficiency; and (5) conducting pilot-scale validation supported by comprehensive techno-economic and environmental assessments to address scale-up challenges, including optimizing large-scale electrochemical cell design and enhancing proton exchange membrane stability;.

The main contribution of the present review is to clarify the scenarios where electrocatalytic and biomass fuel cell technologies show genuine promise and identify the critical limitations that must be resolved before industrial deployment can be realistically considered, thereby providing a comprehensive roadmap for future research in this field.

## Figures and Tables

**Table 2:** Comparative summary of conventional and emerging treatment technologies for pulping and paper-making wastewater.

Technology Category	Main Target Pollutants	Representative Treatment Performance	Main Advantages	Main Limitations	Applicable Wastewater Scenario	Engineering Maturity	Refs.
Physical methods	Fibers, suspended solids, coarse colloids, part of color	Mainly effective as pretreatment or solid–liquid separation; limited for dissolved refractory organics	Simple operation, high practicality, easy integration with downstream units	Poor removal of dissolved COD, lignin, AOX and low-molecular refractory compounds; follow-up treatment is usually needed	Fiber recovery, primary clarification, load equalization, pretreatment of mixed mill effluent	High	[6,68,69,70]
Chemical methods	Color, lignin-derived compounds, AOX, colloids, non-biodegradable organics	Often effective for rapid decolorization and partial COD/AOX reduction	Fast response; useful for toxic or refractory fractions	Chemical consumption, sludge generation, possible secondary pollution, cost sensitivity	High-color bleaching effluent, refractory side streams, pretreatment or polishing	High	[6,68,69,70]
Biological methods	Biodegradable COD/BOD, part of phenolics and soluble organics	Strong for biodegradable organic load removal; aerobic/anaerobic combinations often outperform single-unit systems	Mature, scalable, relatively economical for bulk-flow treatment	Limited for highly toxic, high-color, AOX-rich or strongly refractory streams; sludge and process stability issues remain	Mainstream mill wastewater with moderate biodegradability; secondary treatment trains	High	[6,68,69,70]
Electrochemical methods	Refractory organics, color, lignin fragments, phenolics, AOX precursors	Promising for refractory oxidation and biodegradability enhancement under controlled conditions	Tunable operation; useful for difficult-to-treat wastewater	Energy demand, electrode cost/fouling, electrolyte sensitivity, and scale-up uncertainty	Concentrated or toxic side streams, pretreatment, polishing, coupled processes	Low–medium	[6,70]
Integrated methods	Mixed pollutant loads including COD, color, lignin and toxic fractions	Usually outperform single-unit systems by combining complementary mechanisms	Better overall removal and greater design flexibility	Higher system complexity and more demanding optimization/control	Wastewaters with variable composition or multiple treatment targets	Medium–high	[6,68,69,70]

**Table 3:** Energy balance of selected industrial wastewater treatment processes.

Wastewater Treatment Process	Energy Consumption [kWh/kg COD]	Energy Production [kWh/kg COD]	Refs.
Aerobic Treatment	0.8–1.0	0	[130]
Electrochemical Methods	6.6	0	[131]
Anaerobic Digestion	0.025–0.1	0.2–0.4	[10,132,133]
MFC Technologies	0.02–0.07	Up to 0.17	[134]

**Table 4:** Comparative Summary of Representative DBFC/LFFC Systems for Lignin-Rich Stream.

System	Fuel/Feedstock	Catalyst/Mediator	Peak Power Density	Product/Selectivity	Key Limitations	Ref.
Early liquid-mediator DBFC	Sulfate lignin	Anthraquinone-2-sulfonic acid (AQS), liquid catalyst	0.34 mW/cm^2^	Electricity generation; product selectivity not reported	Very low power density; mediator recovery and recycle not addressed	[135]
POM-mediated DBFC	Lignin	Phosphomolybdic acid (liquid catalyst)	≈5 mW/cm^2^	Electricity generation; Faradaic efficiency improved	Acidic mediator system; liquid catalyst recovery and system integration still required	[136]
Fe-mediated symmetric biomass flow fuel cell	Sugarcane bagasse, rice straw, other biomass residues	Fe^3+^/Fe^2+^ redox couple	54.5 mW/cm^2^	Electricity generation; oxidation to value-added chemicals	Laboratory-scale demonstration; stack integration and mediator circulation remain unresolved	[137]
Direct lignin liquid flow fuel cell	Corn stover alkaline lignin	Ferricyanide/vanadyl(V) redox couples	200.3 mW/cm^2^	Overall electron-transfer efficiency ≈ 90%	Liquid mediator recovery; system complexity; mainly relevant to segregated lignin-rich streams	[138]
Lignin-based flow fuel cell (LFFC)	Sodium lignosulfonate/lignin	CoS@Ni foam (solid electrocatalyst) with VO_2_^+^/VO^2+^ cathodic redox couple	176 mW/cm^2^	Anodic selectivity for lignin oxidation: 97%	Catalyst durability and fouling in real wastewater still need validation	[139]

## Data Availability

No new data were created or analyzed in this study. Data sharing is not applicable to this article.

## Abbreviations

BL	black liquor
AOX	adsorbable organic halides
AOPs	advanced oxidation processes
BEF	bio-electro-Fenton
BESs	bioelectrochemical systems
BOD	biochemical oxygen demand
CCF	chlorine-containing bleaching
COD	chemical oxygen demand
DBFC	direct biomass fuel cell
DCS	dissolved and colloidal substances
EAOPs	electrochemical advanced oxidation processes
ECF	elemental chlorine-free
EF	Electro-Fenton
ER	electrocatalytic reduction
FAO	Food and Agriculture Organization
ISO	International Organization for Standardization
KL	kraft lignin
LFFC	lignin-based flow fuel cell
MEC	microbial electrolysis cell
MFC	microbial fuel cell
P&P	pulping and paper-making
PNSB	purple non-sulfur bacteria
POM	polyoxometalate
SCWO	supercritical water oxidation
SS	suspended solids
SSL	spent sulfite liquor
TCF	totally chlorine-free
TOC	total organic carbon
TRS	total reduced sulfur
TSS	total suspended solids
VOCs	volatile organic compounds
